# A New Sensors-Based Covert Channel on Android

**DOI:** 10.1155/2014/969628

**Published:** 2014-09-14

**Authors:** Ahmed Al-Haiqi, Mahamod Ismail, Rosdiadee Nordin

**Affiliations:** Department of Electrical, Electronic and Systems Engineering, National University of Malaysia (UKM), 43600 Bangi, Malaysia

## Abstract

Covert channels are not new in computing systems, and have been studied since their first definition four decades ago. New platforms invoke thorough investigations to assess their security. Now is the time for Android platform to analyze its security model, in particular the two key principles: process-isolation and the permissions system. Aside from all sorts of malware, one threat proved intractable by current protection solutions, that is, collusion attacks involving two applications communicating over covert channels. Still no universal solution can countermeasure this sort of attack unless the covert channels are known. This paper is an attempt to reveal a new covert channel, not only being specific to smartphones, but also exploiting an unusual resource as a vehicle to carry covert information: sensors data. Accelerometers generate signals that reflect user motions, and malware applications can apparently only read their data. However, if the vibration motor on the device is used properly, programmatically produced vibration patterns can encode stolen data and hence an application can cause discernible effects on acceleration data to be received and decoded by another application. Our evaluations confirmed a real threat where strings of tens of characters could be transmitted errorless if the throughput is reduced to around 2.5–5 bps. The proposed covert channel is very stealthy as no unusual permissions are required and there is no explicit communication between the colluding applications.

## 1. Introduction

In a secure system, data are protected from unauthorized access or modification, and code is protected from unauthorized execution or alteration. In a multiuser environment, this need is obvious where a user's process might attempt to overwrite part of the code in the address space of another user's process. In such a case, enforcing process isolation by the underlying hardware design or operating system can prevent the threat. A process might try as well to access data objects (e.g., read a file) or other software or hardware resources (e.g., execute a program or use an I/O device, resp.) for which it has no corresponding access rights. In these cases, the system should ensure safe operation of all processes, including the untrustworthy ones, by enforcing a proper access control policy. An example of this mechanism is the file permissions in Unix-based systems; objects like devices and sockets are represented as files, and each file is associated with a set of separate access rights to the owner, a group, and the public. All processes that belong to a user have the same privileges of that user. Effectively, the system provides a user-based protection, isolating user resources from one another. The working model of most malware is to compensate for their lack of access rights by trying to escalate their privileges to those of a more powerful user (preferably the root), mainly through exploiting software bugs. And the role of the operating system and any other protection application is to counter these attempts.

In single user systems, like modern smartphones, users can download third party applications that are untrustworthy and potentially adversary to the user's data or the network resources. Android operating system is based on the Linux kernel and makes advantage of Linux process-isolation and user-based protection mechanisms to protect the user from installed applications. Because there is only one user, Android extends the protection system by assigning each application a unique user ID, and hence coming back to the point where there are different users isolated from each other in sandboxes. Applications have limited access to system resources by default, and the wish to use any of predeemed sensitive resources must be explicitly declared so that the operating system can monitor and control the access to those resources. Examples of protected resources are functions related to the camera, Bluetooth, GPS, telephony, and network access. It is pertinent to note here that one of the* un*restricted resources is the set of onboard motion and environment sensors, like the accelerometer and barometer.

Another threat model is represented by Trojan kind of malware, where the malicious software does possess the access rights to a resource based on the (deceived) user belief. The malevolent application here can readily access the otherwise private information and, if it was granted adequate permissions, leak that information outside the secure domain. Traditionally, the countermeasure for such a threat relies on user awareness and specialized protection software to analyze statically or dynamically the structure and/or the behavior of the application in question. Similarly in Android, though the permission system cannot help in situations where the rogue application explicitly asks for certain privileges and uses them, the hope is that Android users will become more aware of the permissions model and therefore wiser at judging the request for suspicious permissions based on the application's functionality and its source. Furthermore, antivirus companies already issued versions of their products specifically for Android, and over-privileged applications are straightforward to pick. Perhaps more effective in the context of ill-intentioned and authorized applications is the research on Android information-flow tracking [1], where sensitive data are tainted and traced through the flow of the application, either statically or dynamically. Whenever a labeled object moves from a private domain to a public domain (e.g., sent to the network), the flow is prevented or logged for further inspection.

Yet another model for malware applications is to avoid the over-privileged status and ask for only a partial set of permissions which is in itself short of doing any harm. In this model, a malicious application needs the collusion of another application on the same system, which has complement permissions to fulfill the attacker objective. For example, one application can take the form of a useful electronic personal manager and has access to private personal information but lacks permissions to network access. This application can invoke no doubt in the user about the safety of his information. If another application with network access but no sensitive data permissions (e.g., news reader) can receive somehow the personal information from the first application, then the combined effect is to leak the user's data without raising suspicion. These two applications (we will call them the source and sink applications, respectively, following the terminology in [2]) should communicate covertly in order to avoid detection by data-flow analyzers. Tainting solutions can conceivably detect flow through* overt* channels, channels meant for communication using data objects by design, like files and shared variables. In Android, these also include interprocess communication mechanisms like intents and broadcasts. In contrast, when using* covert* channels the available flow tracking solutions are much less effective, as confirmed by recent research [2, 3]. Covert channels are means to transfer information, which were neither designed nor perceived as communication channels. Examples of covert channels include file locks, hardware settings, and modulated execution-time delays. If both applications have enough permission to cause and/or note changes in a common resource, it could be very difficult to detect a clever and creative exploit of that channel. In essence, the existence of covert channels subverts Android permission system, allowing an application to extend its capabilities by delegating operations to another conspiring application, all through hidden communication. This is why it is crucial for the integrity of the protected system to uncover as many of such secret channels as possible.

In this paper we reveal one such covert channel on Android platform of relatively low permission requirements and hence more stealthiness compared with other exposed channels so far. This channel depends on the built-in sensors as a vehicle to leak confidential information. The novelty of this channel stems from the fact that onboard sensors are hardly thought of as a possible carrier of custom information, other than those originated from the environment or user activities. Applications cannot generate or directly manipulate sensors data to convey information; they typically read sensors signals passively to obtain a sense of their environment, context, and user actions. However, we show one way in which an application can affect the readings of a specific sensor that is available to all applications, that is, the accelerometer, via the use of vibration feature commonly utilized by notification and alarm applications. We detail one possible design of the attack, comprising the source and sink applications as well as a protocol of communication between them. We also study the channel's bandwidth and error rate.

The rest of this paper is organized as follows. In Section 2 we present the related background and similar research work in order to pave the way for later sections.  Section 3 explains the main idea of the proposed covert channel and dissects the design of the source and the sink applications. In Section 4 we report the setup of our experiments and discuss the evaluation results. We conclude in Section 5.

## 2. Background and Related Work

### 2.1. Covert Channels

Covert channels have been studied for over four decades by now. Lampson first introduced covert channels in the context of the confinement problem [3]: how can a system securely confine host programs so that a service called by a customer cannot leak information to its owner, to use Lampson's terminology. He suggested few rules to ensure confinement or to just bound the capacity of the channel if total confinement is too costly (e.g., it leads to slowing down legitimate operations). To prevent or limit covert channels, they must be detected first. Several works later on proposed non-ad hoc methods to inspect a system and discover as many covert channels as possible in a less or more formal manner. Examples are the Shared Resource Matrix method [4] and the Noninterference approach [5]. The earlier methods were mainly concerned with local covert channels in a single system, typically multiuser mainframe. When computers started to connect to each other in networks, people started to think of network covert channels through which information in a local host is leaked to the owner of a guest process exploiting networking protocol structures. A good survey of network covert channels with countermeasures is [6].

One way to model covert channels is the prisoner problem proposed by Simmons [7]. Two prisoners are planning to escape in the presence of a closely watching warden. To exchange the plan, they must hide the existence of the communication in the first place, and that is where covert channels resemble steganography, in contrast to cryptography. From this perspective, covert channels are a sort of security-by-obscurity approach. Supervising software is mainly concerned with overt channels and would probably overlook information change over a channel it does not know about. That is why the discovery of such channels in new systems is a crucial step towards securing the systems.

Covert channels are often classified as being either timing or storage channels, though other variations with less obvious distinction did also appear. Covert storage channels imply changing a data item, like a configuration setting, by a sender process and reading off the change by another receiver process. Timing channels involve modulating the amount of time, or delays, spent in performing some task or using some resource, detectable and decodable by the receiver. The proposed channel in this work is at first glance hard to classify discriminately, as it stores the leaked data onto sensors signals in form of vibration changes, but relies on measuring the modulated delays of those vibrations. However, we classify our covert channel in the covert storage channels category because there is no need for external clock reference to measure the changes and timing information is included in the sent signal, as described later.

### 2.2. Android Covert Channels

**Figure 1:**
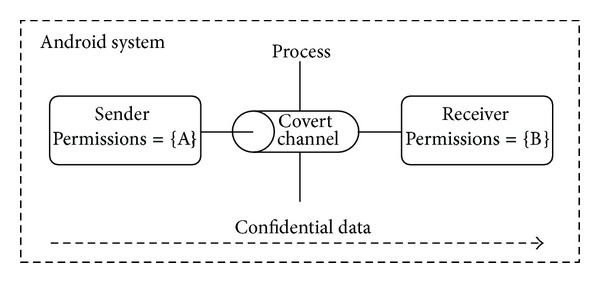
General model of Android covert channel.

Similar to the history of covert channels on traditional computers, the first discussed covert channels on Android platform were local, aiming to overcome the tight monitoring of new protection solutions, like TaintDroid [1] and to avoid the suspicion of asking too many permissions, thus bypassing mainstream security solutions like Lookout [8]. The general working model of those covert channels is illustrated in Figure 1. Assume that the purpose of the attack needs a set of permissions {{*A*}, {*B*}}. One subset (e.g., {*A*}) is of relatively high privileges with access to confidential information but no obvious means to make use of that information, while the other subset is less demanding, such as the permission to access the network. No subset alone can fulfill the attack and raise doubts. If the attacker managed to install or utilize two colluding apps (a very feasible scenario), then each app would ask for an innocuously-looking subset of the permissions and work stealthily, besides doing its public job, on fulfilling its part, either sending or receiving the confidential data over a covert channel that is not known to the system, and hence breaking the process-isolation principle in Android.

Authors of Soundcomber [9] suggested first to use covert channels on Android to leak stolen information to the attacker. Soundcomber extracted small amounts of valuable data (e.g., credit-card numbers) from short recordings of the user's calls at selected portions, but it lacked the permission to access the network, by assumption. The authors then presented two methods to send the sensitive data to Soundcomber's master. The first is by exploiting the browser to embed the data in a requested URL and the second method is to make use of the network access rights of another paired application, called the deliverer, which received the data over four suggested covert channels: vibration setting, volume setting, screen status, and file locks; the first three of which are new and specific to Android platforms.

A more comprehensive list of Android covert channels followed in [2]. The authors implemented and analyzed previously known covert channels, as those utilized by Soundcomber, as well as new covert channels, including the type of exchanged intents, synchronizing the discovery of UNIX sockets availability, enumerating threads number of the paired application, and modulating the time of CPU-usage. The paper included also a set of overt channels like communication over broadcast intents, network sockets or shared preferences, and files. We consider such latter channels outside our interest, as they are potentially prone to be detected by straightforward tainting and flow-tracking techniques. One particularly reported channel, the system log, has indeed been closed by Google since Android API 16, where the READ_LOGS permission is no longer available to third party applications.

A following paper attempted to avoid limitations of performing explicit communications or accessing system files that could be restricted, by introducing new covert channels with more stealthiness at the cost of lower throughput [10]. The authors used changes in the UNIX priority levels of processes to signal the start and end of sending sensitive data, thus producing a cover timing channel. They also used the timing of changes in screen status as well as the life-time of the sending process to convey sensitive data to the receiver.  Table 1 provides a list of the discussed covert channels in the three previous works along with a brief description of each, the reported throughput in their respective conducted scenarios, and the limitations in terms of possible countermeasures using known solutions in the literature.

This list constitutes the complete set of published covert channels on Android so far. For completeness, we also note the attempt to implement an Android network covert channel in [11]. However, the implemented attack is not feasible on nonrooted phones, since Android does not allow the use of raw sockets necessary to manipulate IP interpacket delay times, as acknowledged in the paper.

As notable from Table 1, common techniques of the proposed covert channels include: (1) referring to system settings like vibration or volume settings, (2) referring to a system file such as /sys or /proc files to obtain frequency or usage statistics, and (3) exploiting explicit overt channels as Intents or UNIX sockets. Some of the more sophisticated proposals to monitor intercomponent communication and communication through API calls are capable of detecting and restricting those kinds of techniques. A popular example is XManDroid framework [12]. This solution implements system-centric policy enforcement on the kernel and middleware levels and monitors access to different resources including the file system and all sockets. It is also possible to monitor access to system settings and detect abnormal change patterns relative to the usual or perceptible human behavior, employing a properly chosen threshold. This latter approach was adopted in [13], where the design and implementation of an application-layer detector for covert channels on Android was presented. Further, greedy battery consumption by some techniques (e.g., CPU timing channel in [2] and polling all UNIX processes in [10]) would probably raise the attention of the user or of solutions that address this specific usage anomaly, for example, [14].

Our approach avoids the aforementioned techniques. It does not rely on system settings or system files and does not establish any explicit communication between the sender and receiver applications.

### 2.3. Android Sensors

#### 2.3.1. Background

Android supports a variety of motions, positions, and environmental sensors. It provides a sensor framework that comprises classes and interfaces through which developers can access onboard sensors [15]. Two main tasks to accomplish using the framework are to identify available sensors and their capabilities and to monitor sensors events. For example,* Sensor* class has methods to report a specific sensor's type, vendor, resolution, power, and minimum delay. Another class,* SensorEvent*, supplies raw sensors samples along their corresponding timestamps. An application can register with one or more sensor-event listener, asking the operating system to update it with sensors reading at a preferred rate. Access to the built-in sensors is not restricted by any particular permission, up to this writing. Sensors like accelerometers and magnetometers are almost standard components on modern Android powered devices and gyroscopes are catching closely, while ambient sensors such as barometers and thermometers are fewer. Recently, Google has even extended Android to support wearable sensors through Android Wear [16]. From among all those sensors we utilize the accelerometer in our covert channel, due to its sensitivity to vibration.

**Figure 2:**
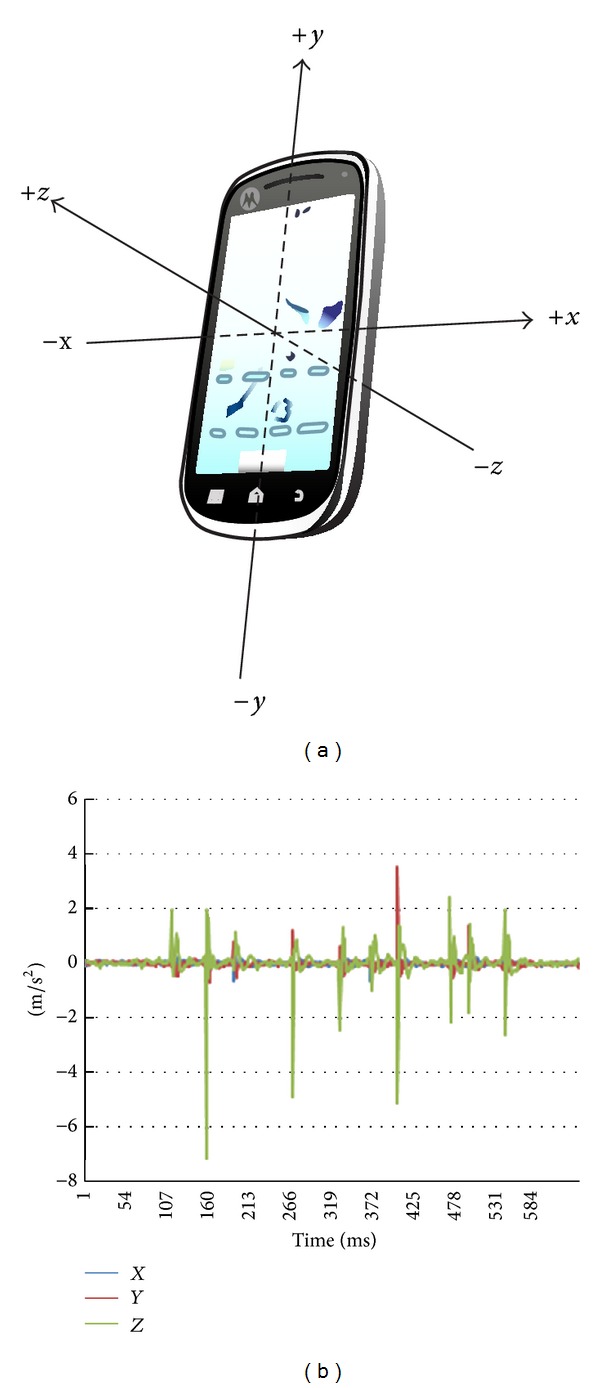
(a) Coordinate system of the accelerometer. (b) Sample of accelerometer readings showing the effect of typing the digits “0123034880.”

Accelerometer readings are expressed with reference to a 3-axis coordinate system. This coordinate system is defined relative to the device's screen when the device is held in its default orientation (Figure 2): the *x* axis is horizontal and points to the right, the *y* axis is vertical and points up, and the *z* axis points toward the outside of the screen face. In terms of structure, those data are delivered in the form of a multidimensional array named values. Elements of the array correspond to sensor samples along each of the three coordinate axes. For example, values[0] would convey the acceleration force (change in velocity with respect to time) along the *x* axis while values[1] contain the acceleration force along the *y* axis, and so forth, all in meters per second square. A separate variable contains the timestamp of each particular sample.

#### 2.3.2. Sensors-Based Threats

More traditional sensors like GPS, camera, and microphone have received earlier attention on smartphones in general including Android phones (e.g., [17]). Later on, researchers started to focus on sensors-based privacy and security issues in two directions. On one hand, less-traditional sensors such as motion sensors proved to be a real threat in ways nobody concerned before. For example, few works have demonstrated the feasibility of inferring keystrokes on Android touch screens depending on the accelerometer and gyroscope (e.g., [18–22]). Accelerometers have been even used recently as a source of fingerprints to identify Android devices remotely [23]. On the other hand, directing conventional sensors again, new innovative techniques have been devised to breach users' privacy or security. Cameras, for instance, were exploited to track the user's eye gazes while typing sensitive data and then employ image processing methods to figure out the data being typed [24]. Soundcomber [9], which suggested the first covert channels on Android, actually addressed an inference attack employing the onboard microphone as the main target vector and only suggested the covert channels as a possible solution to escape the tight governance of security mechanisms.

In this paper, we propose, for the first time using built-in sensors, the accelerometer, in particular, as a vehicle to communicate covertly between Android Trojan applications. All previous attacks have dealt with the accelerometer as a mirror to reflect users' actions (e.g., typing), but we here suggest to use the vibration motor found in Android phones to manipulate sensors output and reflect input from other applications.

## 3. Vibrate-and-Sense Covert Channel

Assuming the general model depicted in Figure 1 where two apps are trying to complement their respective permission sets and communicate covertly and calling the app with access to high-sensitivity data* the source*, whereas the app to disclose those data* the sink*, we restate the minimum requirements for a covert channel to exist, originally listed in [4] as follows.

- The source and sink must have access to a shared resource.
- There must be some means by which the source can force the shared resource to change.
- There must be some means by which the sink can detect the resource change.
- There must be some mechanism to initiate the communication between the source and the sink and for sequencing the events correctly.

In the following subsections we address these criteria presenting the design of our covert channel, including the fundamental concept underpinning its covert communication and the algorithms of our malware (i.e., the source and sink apps), as well as the offline methods that could be applied by the attacker to extract the useful information.

### 3.1. The Concept

The main idea behind the new covert channel is to exploit a shared resource to which all applications have free access, that is, sensors data. This fulfills the first condition in the list of requirements above. What makes this channel so stealthy is the fact that there is no obvious way to meet the second condition. Apparently readings from sensors are generated by hardware chips caused by external influences from user actions or the environment, and applications have no control over signals from sensors (i.e., they cannot enforce the shared resource to change) unless they have control of user motion or the environment, which is hard to imagine.

**Figure 3:**
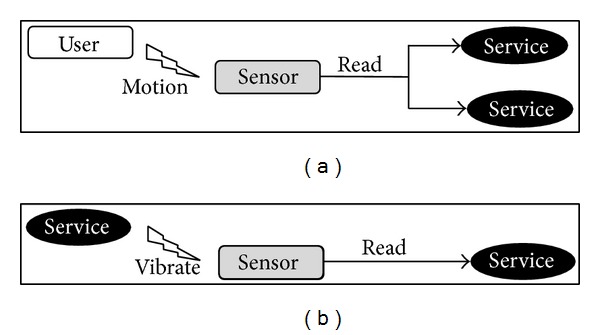
(a) Normal model of reading sensor signals caused by user actions. (b) Exploiting the vibration function to modulate sensor signals and establish a covert channel.

Yet, we observe one feature in Android devices that can actually affect sensors in a programmatically-controlled manner. The vibration motor common in modern phones is available for applications as a tool to notify users of events, but applications exist that even use this feature for massage! Vibrations could be controlled in duration and number, so that a pattern of vibrations can be used to encode a hidden message. Vibration codes cannot be received, in the usual sense, by other applications, as vibration is not data but is motion. Nevertheless, these motion patterns might affect the “base” motion of the phone and hence modulate signals from sensors that can sense and report motion. Some technique of “demodulation” would be required to decode the hidden message and extract the stolen information. The concept of two applications (implemented here as services) communicating covertly through the vibrate-and-sense mechanism is illustrated in Figure 3.

The two communicating services are parts of two Trojan applications installed by the user from the same source, or in some way under the control of the attacker. This assumption is very viable considering the large number of developers who publish several applications and the many ways in which a follow-up application could be requested (e.g., as an add-on). It is also possible to think of groups of attackers colluding to distribute parts of malware, with illusively useful purposes, to serve as either covert sources or sinks.

### 3.2. The Sender Application

The sender application must have two capabilities: the access to sensitive data and the ability to vibrate the device. Both capacities can be granted through appropriate permissions given a convincing Trojan function. For example, an application to manage calendar events, text messages, and phone calls with appropriate vibration notifications and alarms is a common and legitimate application function. We have found successful applications in Google Play that ask much more permissions for similar purposes, sometimes including the full network access, for which we assume no permission.

**Figure 4:**
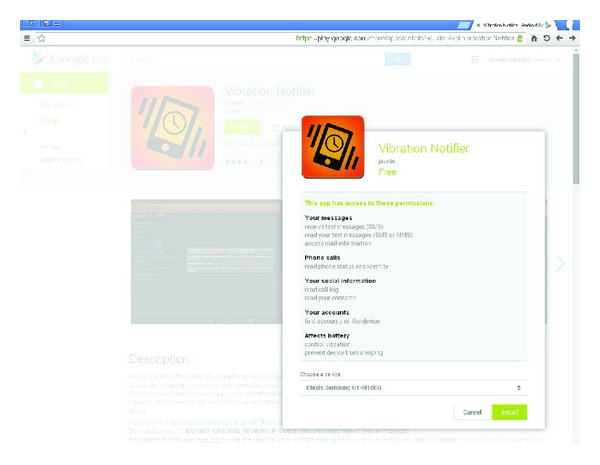
Example of a real benign app in Google Play with its list of requested permissions, which is a superset of the permissions required for our source application.

Many Android phones suffer from halting the sensor readings when the screen goes off, which was actually reported in the Android issue-tracking system since 2009 [25]. The most common workaround among developers is to utilize a partial* wake lock* to keep the screen on while sensing is needed. All-time-sensing applications face the issue of battery drain, as a result of preventing the phone from sleeping continuously, but turning on the screen to communicate covertly for a short while is not a matter. All the application has to do is to ask for an extra permission to hold a wake lock, which is a common permission for a wide variety of tasks (e.g., media playing, online downloads and updates, sensing, and visual notifications). Therefore, we chose to adopt this solution because we need to exchange covert data during idle times, preferably deep in the night, for a better stealthiness. We could also have assigned the task of awakening the screen to the sink application, for example, justified by the need to keep the screen working while reading the collected news, assuming a “news reader” sink application. Indeed, we can find innocent applications in Google online store with all permissions discussed above and more. An example application with similar functionality to our sender malware is “Vibration Notifier,” whose list of requested permissions is shown in Figure 4. The permission to hold a wake lock is described as “prevent device from sleeping.” It might be interesting to note that the author of the application assures in the description area that no personal data is read despite the seemingly excess permissions. As a proof of his claim, he explicitly notices that the application cannot access the network, so that even in the untrue case of reading off private information, none might be leaked outside the device. In fact, this is a nice real-world demonstration of the scenario we assume in this paper, as well as in most of the papers addressing covert channels on Android.

The source application is working as follows. Whenever the application has some data to leak, it starts a service via an* intent* and passes the data within the intent. This started background service is known to the user and is an essential part of the application functionality, as the intended functions need to watch for events even after the main interface activity is destroyed. The user can explicitly stop the service, but the application can always start the service each time it is launched by the user or by a service-issued notification, sending another piece of stolen data in the intent, if any. The service checks for data in the intent and registers for screen events (turning on/off) if there are received data. The service would receive an* ACTION_SCREEN_OFF* broadcast action every time the screen goes off. The service also has to implement a broadcast receiver method* onRecive()* where it examines whether the data have already been sent. If not, it forks off a thread that actually establishes a covert channel through several steps.

**Figure 5:**
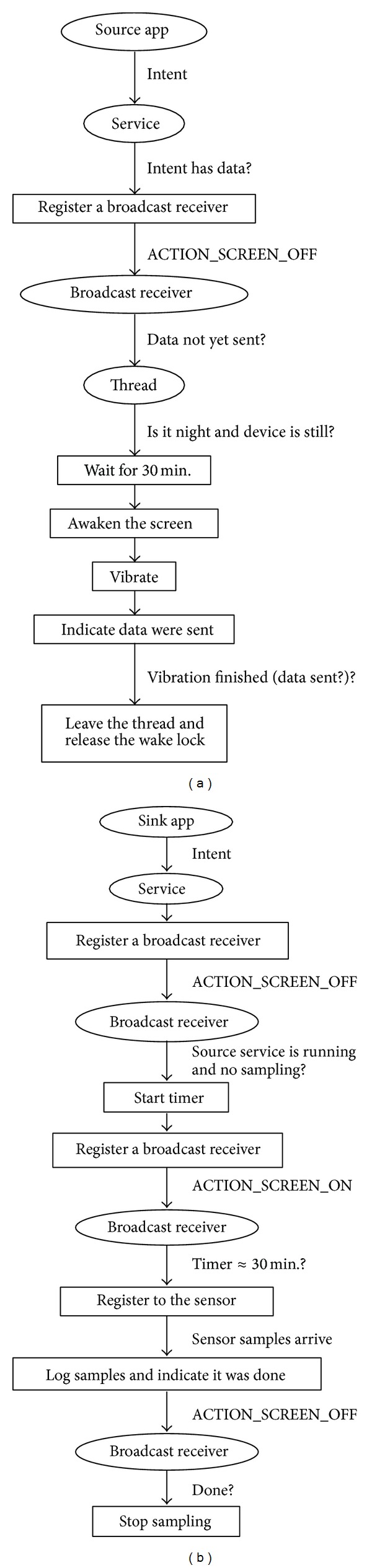
Logic flows in algorithms of the (a) source application and (b) sink application.

First, the thread attempts to ensure that the phone is still and the user is away for maximum secrecy. It makes sure the accelerometer signals are stable and the time is night and further waits for a predetermined and agreed upon period of time (e.g., 30 minutes) to ensure that the user has left the phone. Next, the thread acquires a wake lock and turns the screen on to enable sensor samples and to signal the receiver to start reading accelerometer data. It then vibrates according to a pattern that matches the encoded data. After that, a special flag is set indicating that the data was sent, so that if the screen went off again this cycle is not repeated until the service is started the next time with new data to send. Finally, sufficient time is given for the vibration to complete, then the thread is left and the wake lock is released. This algorithm is shown if Figure 5(a).

### 3.3. Data Encoding

Android provides an abstract class,* Vibrator*, to operate the vibrator [26]. The* Vibrator* class includes two overloaded methods, each is called* vibrate()*. The first method causes the device to vibrate constantly for a specified amount of time in milliseconds, passed as a parameter. The second method gathers more than one period of vibrations into a single pattern of alternate periods, in milliseconds, and turns the vibrator alternately on and off during those periods. This pattern of alternate vibrations is passed as an array of the type* long*. The first element is the number of milliseconds to wait before starting the alternate on/off vibrations. For instance, the pattern *p* = {0,1000,2000,2000,1000,3000,2000,4000} indicates immediate vibration for 1000 ms without any initial delay. Then 2000 ms of no vibrations follows, and so forth. The last element in this pattern is a vibration for 4000 ms.

In this paper, we are using the method variation of vibration patterns to encode bits of exchanged data. The level of vibration intensity cannot be changed, leaving us with a binary signal of on and off values. Following a simple NRZ encoding scheme, the on state would represent a bit of value “1” and the off state would symbolize a bit value of “0.” Depending on the chosen interval of a single bit the bandwidth of this transmission channel can be calculated. The smaller the bit interval (i.e., the shorter the basic vibration period) the more bits that could be encoded in one second and hence the more the bandwidth. We would ideally prefer channels with high capacity to increase the throughput. However, if the vibrator periods are too short, we might not be able to extract the bits out of the accelerometer signal, especially in cases of long strings of zeros or ones. Therefore, it is important to select a proper bit interval that can be reflected by a distinguishable vibration period.

**Figure 6:**
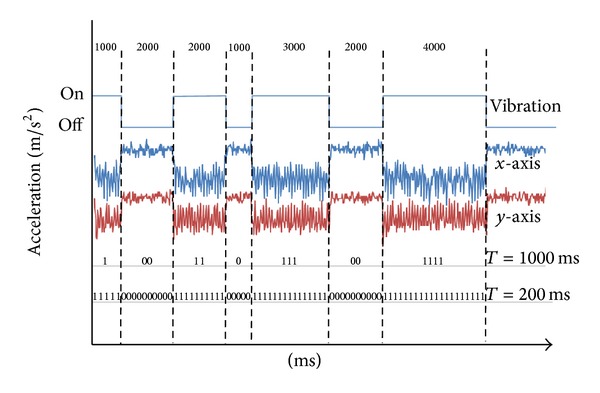
Encoding bits into vibration patterns. The accelerometer raw signals along the *x* and *y* axes are also shown affected by the modulating vibrations. *T* = bit interval.

As an example, consider the pattern array given above. If we chose a bit interval of 1000 ms, then the vibrator would virtually reveal 15 bits' worth of data. On the other hand, the vibration pattern would carry 75 bits if the bit intervals were 200 ms. In the former case the bandwidth is 1 bit/s while in the latter it is 5 bits/s.  Figure 6 presents a visualization of this example pattern, its impact on the accelerometer data when transferred to vibrations, and the corresponding bit sequence based on the chosen bit interval. During the vibrations, sensor readings are more variant w.r.t. their corresponding mean and more negative in the *x* and *y* dimensions relative to their respective values with no vibrations, in this particular case.

In malware applications where the target data are numeric (e.g., GPS coordinates), there is no need for character encoding and bits of the numeric type are sent to the receiver. In our malware, we are mainly concerned with strings of characters (e.g., last SMS received). Therefore, we need to translate characters into sequences of bits using some encoding scheme. We could adopt a variable-length code like Huffman code to increase transmission throughput, but for simplicity we use the basic ASCII code, excluding the extended codes, to represent each character using 7 bits. It is those bits that are encoded as vibration patterns and sent by the source application to the sink via accelerometer data. An example of encoding the email address “ahmadalhaiqi@gmail.com” is shown in Table 2.

### 3.4. The Sink Application

Similar to the source, the sink application should appear benign to the user with a useful purpose. The only permission we require for the sink is the* Internet* access permission. There are numerous applications whose function depends on the access to the network. The example we selected for our implementation is a news reader that collects news feeds from several online resources and presents them locally for review and reading. As an extra benefit, this sort of application would also allow us to request the permission for holding wake locks on the screen, had we failed to find a convincing reason to ask for this permission in the source. The main real purpose of the sink, however, is to receive transmitted data from the source via accelerometer readings. Whenever the sink receives some data in the form of sensor signals, it can either process those data online (on the device) and extract the encoded secrets, or send the whole set of sensor data in a file to the attacker for offline processing. This is a design decision that depends on several factors.

On one side, performing the processing online is more attractive as it leaves only small amount of valuable data to be transmitted through the network, leading to hardly noticeable bandwidth use. On the other side, if the employed techniques to extract the data are computationally heavy, attention might be raised either by unusual resources usage or suspicious energy consumption. Also, offline processing might take advantage of the available powerful computation packages on traditional platforms, while handling complicated tasks on the phone might probably necessitate writing custom code from scratch. In our case, the sink collects sensor readings only during the covert communication, so that the accumulated file size in each session is only in the order of few kilobytes for a short string message (e.g., a short SMS or a contact name). Hence, the sink just sends the collected data in a* comma separated values* (CSV) formatted file through the network.

Details of the sink algorithm are outlined in Figure 5(b). In a similar reasoning to the case of the source application, whenever the sink is started or brought to the front it would create or restart a service. The service immediately registers a broadcast receiver for screen events and waits for the screen to turn off. The screen state in our design is not used to convey covert data; it is rather a separate control channel to help synchronize the two processes and minimize the time during which the sink has to sample sensor data. This is one reason why the proposed covert channel is very stealthy and lightweight compared to other methods. Upon receiving an* ACTION_SCREEN_OFF* broadcast, the sink service checks for two conditions. First, it makes sure that sampling the accelerometer is not actually going on (in which case it would have already been receiving some data). Second, it ensures that the source service is currently running through the* getRunningServices()* method. If both conditions are met, it starts a timer and registers a broadcast receiver for the screen turn-on event. Please remember that the source service is going to turn on the screen when it is about to send some data. Upon the* ACTION_SCREEN_ON* broadcast, the next step is to test the timer value. This test is necessary because the screen might be turned on by the user or other applications. If the timer value is the stipulated 30 minutes, then the sink service starts reading the accelerometer data logging them in an internal file of its own. Only after that if the screen goes off, the service stops sampling and proceeds to extracting the private message or just sending the whole acceleration record to the attacker.

### 3.5. Message Detection and Extraction

**Figure 7:**
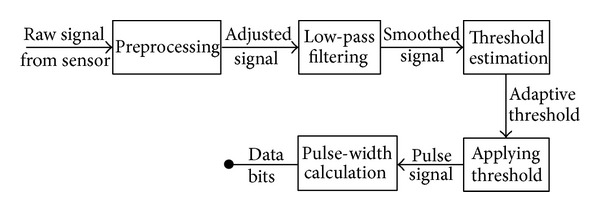
The principle framework to detect and extract vibration patterns and subsequently the hidden message bits from raw accelerometer data.

**Figure 8:**
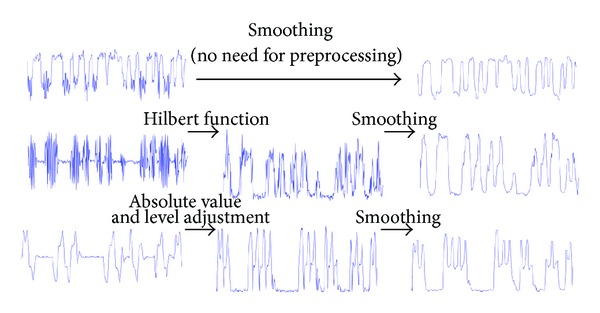
Different vibration patterns need different preprocessing approaches. The typical shapes we encountered during our evaluation of the proposed covert channel are presented below. In the first case no preprocessing is needed as the vibrations are easily distinguishable from the rest of acceleration samples at rest.

To retrieve the transmitted information, the vibration pattern must be extracted out of the accelerometer data. There are several methods to follow, depending on the complexity of the task, ranging from basic signal processing techniques to complicated pattern and change detection algorithms. We propose a general framework as a basic methodology to detect and extract the vibrations, presented in Figure 7. Throughout our initial investigation of the introduced covert channel, we found no specific universal technique to deal with all various effects of vibration patterns on acceleration data. In particular, the preprocessing step in our general framework can vary according to the particular shape of the combination of vibration and acceleration at no motion. Samples of the typical three variations are shown in Figure 8. In the same figure, we also show the specific three approaches we implemented in our experiments: either (1) no preprocessing is needed, (2) extracting the envelope, or (3) moving all spikes to one side and adjusting the level of the signal. This variety of vibration shapes is probably due to different types of vibration motors, their position within the phone body, and the form factor of the phone itself.

For example, referring to Figure 6, the portions of the signal associated with vibration is easily distinguished from the rest of acceleration data when the device is still. From the case in this figure, it seems that the detection process lends itself to a straightforward comparison task against a threshold. We found that simple smoothing of the signal before the detection is helpful to reduce high variance (and spikes) on both sides of the on and off regions on the signal. This is especially obvious in the portions of the signal where the vibrator is on. Following this approach, we do not exploit the difference in the variance to tell the two regions apart, but we rather suppress those variations in favor of exploiting the difference in the signal levels on each side.

**Figure 9:**
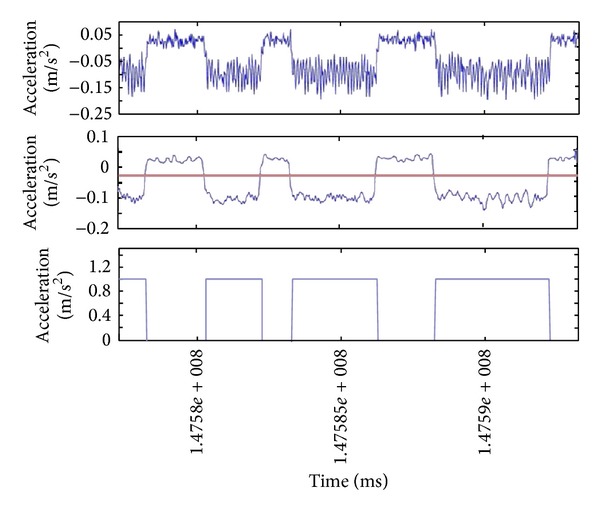
Example of the simple process to smooth the accelerometer signal and applying a dynamic threshold to produce a pulse train in which the width of each pulse is the delay of one of two alternate vibration states, on or off. The hidden message is encoded in those delays: the on state represents one or more 1 bit and off state stands for one or more 0 bits.

Detection of the vibration parts is only the first step towards the extraction of the encoded message. The message is encoded in the time delays of the alternate areas of vibration, therefore the next step is to calculate the width of each region and estimate the number of enclosed bits. For that purpose, we apply a dynamic threshold (determined based on the average or variance of the signal) to the detected pieces of on/off vibrations and produce a series of pulses. Each pulse constitutes one or more “1” bit, according to the interval of each bit; the remaining areas are those corresponding to “0” bits. Knowing that each character is 7 bits long, the original message could be constructed back. This method does not require synchronization between the source and the sink services as the timing information is included within the accelerometer data. Had we been able to synchronize the two processes precisely, then the simpler technique of sampling the signal at the middle of each bit interval would reveal the bit sequence, relying again on a suitably selected threshold value. These steps are depicted in Figure 9 for the example bit sequence and corresponding vibration pattern in Figure 6.

The described method is quite reliable when proper smoothing and the correct threshold are applied. Flipped bits errors are of localized impact as every 7 bits symbolize only one character. However, missing even a single bit altogether, as a result of inadequate bit interval, would affect all succeeding encoded characters. Either sufficiently long bit intervals should be chosen or some error detection/correction mechanism might be incorporated in the data to reduce or eliminate bit errors. Both options translate to a reduction in the channel throughput, which we trade in, by design, for a more robust and stealthy covert communication, given the typical small amount of valuable information.

## 4. Experimental Setup and Evaluation

Our main goal in evaluating the proposed covert channel is to address the question: can vibrations really cause discernible effects on sensors signal? And if so, can the process described earlier actually pick out the sent vibration patterns and decode them into the stolen data bits? It is also important to notice how far can we go in packing more bits in vibrations spanning a given time interval; in other words, what is the expected bandwidth capacity of the proposed channel. This latter question cannot be directed in isolation of the associated error rates, so we condition our estimation of bandwidth capacity on error-free data transmission between the source and sink services.

**Figure 10:**
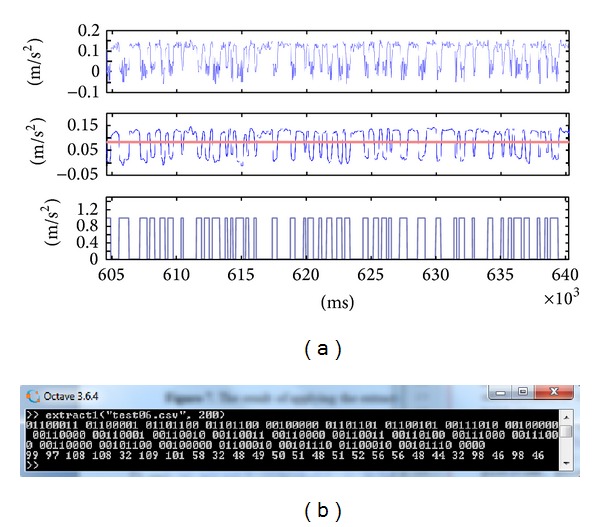
(a) The result of applying the extraction method on modulated acceleration signals from Samsung S2. (b) The decoded bit string and equivalent 7-bit ASCII codes.

**Figure 11:**
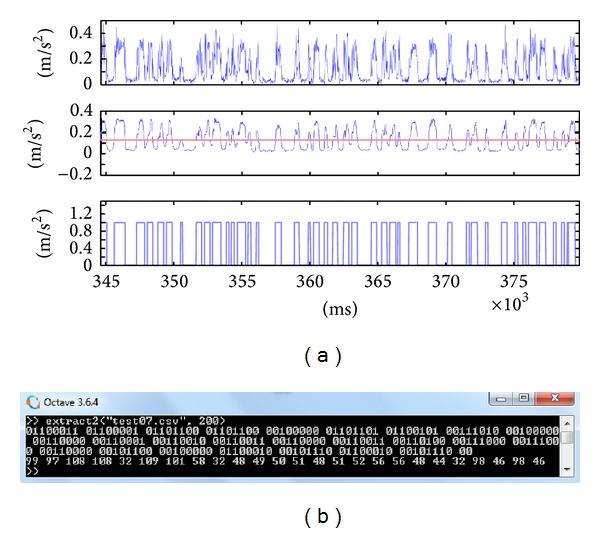
(a) The result of applying the extraction method on modulated acceleration signals from Samsung S3 Mini. (b) The decoded bits and equivalent 7-bit ASCII codes.

**Figure 12:**
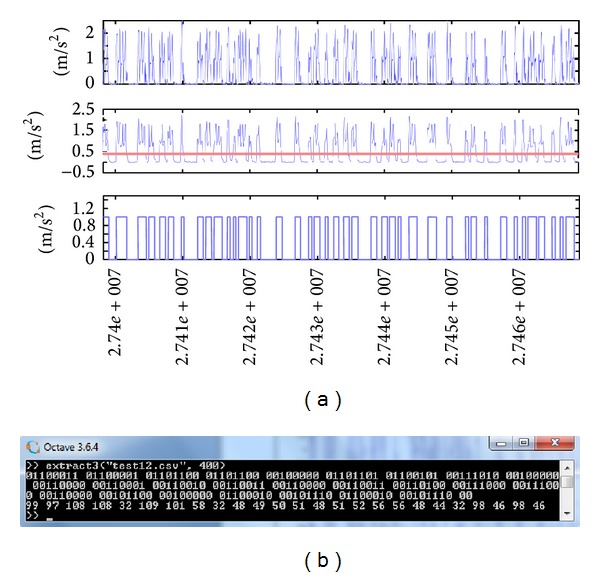
(a) The result of applying the extraction method on modulated acceleration signals from Samsung S3. (b) The decoded bits and equivalent 7-bit ASCII codes.

In order to answer the above questions we implemented our malware in two separate Android apps, the source and the sink. After preliminary examination of the vibration effects on few Android devices, we selected three particular phones on which we installed the malware and evaluated the covert channel performance. The subject test smartphones are of three different sizes and weights: Samsung Galaxy S3, Samsung Galaxy S2, and Samsung Galaxy S3 mini. Using the correct bit interval, we found that bits could be indeed encoded in and then decoded out of the vibrations in the three phones. The results of extracting the hidden bits from accelerometer data in each of the test samples are given in Figures 10, 11, and 12. As discussed earlier, different preparations suit different vibration shapes, but the complete set of bits can indeed be obtained back if the correct technique and bit interval are used.

In all cases of Figures 10–12, the hidden message was a character string, 25 bytes long, supposedly a short SMS. The message text, its ASCII code, and the binary representation are as follows:

 “call me back: 0123034880, b.b.”
 99 97 108 108 32 109 101 58 32 48 49 50 51 48 51 52 56 56 48 44 32 98 46 98 46
 01100011 01100001 01101100 01101100 00100000 01101101 01100101 00111010 00100000 00110000 00110001 00110010 00110011 00110000 00110011 00110100 00111000 00111000 00110000 00101100 00100000 01100010 00101110 01100010 00101110

We could not achieve an error-free transmission with bit intervals less than 100 ms using the adopted methods. For the smaller sized S2 and S3 mini phones, a 200 ms-delay of vibrations for each bit was completely adequate to separate back every bit. However, for the larger S3 phone, we had to increase the bit interval to 400 ms for 0% error rate. That is, the throughput of the heavier device is 1000 ms/400 ms/bit = 2.5 bits per second, in contrast to a 5 bps throughput for lighter devices. The set of 175 bits comprising the 25-character message takes 70 and 35 seconds to be sent, respectively.

The list of the tested phones, their dimensions, weights, and corresponding performances are shown in Table 3.

No attempt was made to include error detection or correction bits, though the concept is feasible and would probably help improve the throughput in the case of larger phones and longer messages. Performance of the test devices was almost the same when placed on a hard surface (e.g., top of a bare table) or a pile of papers. Our experiments also revealed negligible effects of dressing the phones in plastic cases.

In most instances, the acceleration along the *x*-axis yielded the best results, though in few trials, especially in the case of the heavier device, readings of the *z*-axis were easier to notice and extract the vibrations from. When the phone is placed on its back, facing the sky with the front side, vibrations on the *x*-axis are horizontal along the shortest edges, while those on the *z*-axis are vertical away from the ground. Thus, a possible explanation for the inferior performance of the samples on the *x*-axis in heavy phones is that the vibration motor cannot shake the body of the phone sufficiently as with the lighter devices, and the vibrations are confined and manifest in the up-and-down direction. Further, in some of our experiments, the product of the two signals in the *x* and *y* axes gives a very clear output and performs better than any signal on just a single dimension. In real situations, if the attacker preferred to try different possible combinations to find the optimal signal, he can include a sample test message in the initial transmission over the covert channel and experiment with decoding his own known message to reach at the best set of parameters, in terms of the signal axes, bit interval, and extraction technique.

During all of our experiments, we set the sampling frequency of accelerometer to around 50 Hz, equivalent to a delay of 20 ms between each two consecutive samples. This rate, however, is hardware dependent and might not be supported by some devices. For example, the Galaxy S2 phone could endure a minimum delay of 23 ms between the samples.

The detection and extraction algorithms were implemented using Octave scripts. Acceleration data are received from the infected mobile phone through the network in a* CSV* file format and fed as an argument to an Octave function along the desired bit interval. Utilizing the powerful signal processing and vector arithmetic capabilities of Octave is an important advantage of offline processing.

Finally, out of the available commodity sensors on Android devices, we found the accelerometer to be exclusively effective in the context of the proposed covert channel. Gyroscopes did not contribute to encouraging results in our experiments, so we focused solely on the accelerometer. Nonetheless, we expect the gyroscope to be of greater use in the unconsidered situations where the phone is placed in a free-to-rotate position. Typically, the phone has more freedom to rotate when held in hands or on a very soft cushion. Gyroscopes measure the angular rotation speed in radians per second and in our experimental scenarios we assume an idle phone on a stable surface and not in the hands of a user, in order to keep the attack as stealthy as possible. Other motion sensors are by their nature inferior to the accelerometer as well, with respect to this covert channel.

## 5. Conclusions

Motion sensors like accelerometers are hardly perceived as channels to exchange information other than that caused by user actions. Previously, malware applications were suggested to sniff sensors data during user input on touch screens and infer sensitive information. This is a one-way communication from the sensor to the application. We propose in this paper a mechanism to transmit private data the other way around, from a malicious application to the sensor. Because sensors readings are available to all Android applications free of security permissions, any information carried on sensors data can be received by other applications currently running on the phone. The trick is for an application to exploit the onboard vibrator motor to generate patterns of vibrations that encode sensitive data. The vibrations modulate acceleration signals and are intercepted and decoded by another rogue application; the result is a stealthy new covert channel on Android platform. Our preliminary investigations, supported by proof-of-concept implementation confirm the applicability of the attack, and found for an especially difficult-to-detect covert channel. No connection is established between the colluding applications and no particularly suspicious permissions are required other than the common permissions to control the vibrator and to keep the device from sleeping.

It is important to note that the main aim in this paper is to introduce the concept of the new covert channel and provide a taste of the algorithms to use and the results to expect when exploiting the proposed idea. Stealthiness and lightness overweigh channel capacity in our design because much of the private data are produced on a daily basis, and it is essential for the covert channel to last undetected as long as possible, rather than to send as much data as possible at the risk of detection and elimination. It is conceivable that more sophisticated signal processing techniques and perhaps machine learning methods can achieve more channel capacity. It is also possible to extend the study over wider range of Android devices and discover new patterns of techniques for new vibration shapes. All in all, this work is another testimony that collusion attacks on Android exploiting novel covert channels are still an open research problem.

## Figures and Tables

**Table 1:** Summary of Android covert channels in the literature.

Covert channel	Description	Reported throughput (bps)	Possible countermeasures	Ref.
URL	To embed the leaked data into requested URLs through the browser	—	Taint-tracking solutions (e.g. TaintDroid)	[9]
Vibration setting	Sensitive data are encoded in a sequence of changes to the three possible vibration settings (on, off, or silent only)	87	(1) Applying thresholds based on physical limit or behavioral patterns of human interaction (2) Advanced monitoring solutions (e.g. XManDroid)	
Volume setting	Sensitive data are encoded in a sequence of changes to the eight possible volume settings	150	Applying thresholds based on physical limit or behavioral patterns of human interaction	
Screen status	Sensitive data are encoded in a sequence of changes to the two possible screen settings (ON, OFF)	5.29	Applying thresholds based on physical limit or behavioral patterns of human interaction	
File locks	Data are sent through competing for read/write locks on a shared file in a synchronized manner	685	Advanced monitoring solutions (e.g. XManDroid)	

Type of intents	Stolen data are encoded in intent parameters rather than its payload	3216.74–4879.45	Advanced monitoring solutions (e.g. XManDroid)	[2]
Automatic intents	Stolen data are encoded in the automatic broadcasts triggered by changing settings rather than in the settings themselves	50.97–91.06	Advanced monitoring solutions (e.g. XManDroid)	
UNIX socket discovery	Stolen data are encoded in the state (open/closed) of a socket, synchronized by the state of another socket	1818.48–2305.67	Advanced monitoring solutions (e.g. XManDroid)	
Multiple settings	Data are exchanged utilizing many of the system settings at once, an improvement over using only a single system setting like volume level	230.35–286.81	(1) Applying thresholds based on physical limit or behavioral patterns of human interaction (2) Advanced monitoring solutions (e.g. XManDroid)	
Single setting	Similar to the channel of vibration/volume/screen settings above	46.57–66.62	(1) Applying thresholds based on physical limit or behavioral patterns of human interaction (2) Advanced monitoring solutions (e.g. XManDroid)	
Threads enumeration	Sensitive data are encoded in the number of threads spawned by the source application	146.79–156.76	Advanced monitoring solutions (e.g. XManDroid)	
Free space on file system	Sensitive data are encoded in the number of blocks written to or deleted from the disk	9.80–13.07	—	
/proc/stat statistics	Bits of stolen data are conveyed by affecting the number of jiffies used by all processes, which can be read from a usage statistics file	3.26–7.82	Advanced monitoring solutions (e.g. XManDroid)	
CPU timing	Stolen data are conveyed by varying the load on the system so that the receiver process spends more time completing tasks	3.70	Energy-use anomaly detection (e.g. [14])	
CPU frequency	Stolen data are conveyed via change to processor frequency caused by the source and queried from the system, assuming support for dynamic frequency scaling	0.56–4.88	(1) Advanced monitoring solutions (e.g. XManDroid) (2) Energy-use anomaly detection (e.g. [14])	

Task list with screen synch.	Sensitive data are encoded in the time elapsed before the source application kills itself and disappears from the list of running tasks, starting from the point the screen goes off	—	—	[10]
Process priority with screen synch.	Sensitive data are encoded in the time during which the source process changes its UNIX priority level to a predetermined value, synchronized by the change of the screen status	0.65	Advanced monitoring solutions (e.g. XManDroid)	
Process priority	Same as previous channel but without control from the screen status, leading the receiver to continuously monitor processes' priorities	17.6	(1) Advanced monitoring solutions (e.g. XManDroid) (2) Energy-use anomaly detection (e.g. [14])	
Screen status	The screen is watched till it turns off and then the source turns it on, waits until it is turned off again, and encodes the data in the delay before turning the screen on for the second time	0.22	—	

**Table 2:** Example of encoding from a character string up to the vibration pattern. The bit vibration interval is 200 ms and the initial delay is 0.

Target string	ahmadalhaiqi@gmail.com										

7-bit ASCII code	a	h	m	a	d	a	l	h	a	i	q
	1100001	1101000	1101101	1100001	1100100	1100001	1101100	1101000	1100001	1101001	1110001
	i	@	g	m	a	i	l	.	c	o	m
	1101001	1000000	1100111	1101101	1100001	1101001	1101100	0101110	1100011	1101111	1101101

Vibration pattern array	{0, 400, 800, 600, 200, 200, 600, 400, 200, 400, 200, 600, 800, 600, 400, 200, 400, 400, 800, 600, 200, 400, 400, 400, 200, 200, 600, 400, 800, 600, 200, 200, 400, 800, 600, 600, 200, 200, 400, 400, 1200, 400, 400, 1000, 200, 400, 200, 600, 800, 600, 200, 200, 400, 600, 200, 400, 600, 200, 200, 600, 200, 400, 600, 800, 200, 1200, 200, 400, 200, 200}										

**Table 3:** List of tested phones with specifications and corresponding throughputs.

Phone	Dimensions	Weight	Bit interval	Throughput
Samsung I8190 Galaxy S III mini	121.6 × 63 × 9.9 mm	111.5 g	200 ms	5 bps
Samsung I9100 Galaxy S II	125.3 × 66.1 × 8.5 mm	116 g	200 ms	5 bps
Samsung I9300 Galaxy S III	136.6 × 70.6 × 8.6 mm	133 g	400 ms	2.5 bps
